# Paroxysmal Nocturnal Hemoglobinuria: A Diagnostic “Zero-Sum-Game”

**DOI:** 10.7759/cureus.11956

**Published:** 2020-12-07

**Authors:** Qaisar Farooq, Muhammad W Saleem, Zakir Ullah Khan, Niktash Hadi

**Affiliations:** 1 Internal Medicine, Hayatabad Medical Complex Peshawar, Peshawar, PAK

**Keywords:** anemia, hemolytic, hemoglobinuria, paroxysmal

## Abstract

Paroxysmal nocturnal hemoglobinuria (PNH) is a rare intravascular hemolytic anemia caused by an acquired mutation in the phosphatidylinositol-N-acetylglucosaminyltransferase-subunit-A (PIG-A) gene. This mutation leads to the deficiency of cellular anchors for complement inhibitor proteins cluster of differentiation (CD)55 and CD59, predisposing red blood cells to hemolysis by the complement system. We describe the case of a 28-year-old male who presented to the Medical A Ward, Hayatabad Medical Complex, Peshawar, Pakistan, in August 2017 for anemia workup and was later diagnosed as PNH. Current treatment guidelines recommend the use of eculizumab for treating PNH, but the cost and availability of this treatment is a major limiting factor in our resource-poor setting.

## Introduction

Paroxysmal nocturnal hemoglobinuria (PNH) is an acquired form of intravascular hemolysis having a worldwide incidence of five to six cases per million and a prevalence of 16 cases per million. The mean age at diagnosis is 50.0 years with a standard deviation of 18.6. The disease has a slightly higher predisposition for females with 52.1% of diagnosed patients being women [1]. Although the literature contains a lot of information on PNH, the disease is rarely encountered in clinical practice. Moreover, despite thrombosis being a well-known complication of this condition, very few cases of arterial thrombosis have been documented in the literature. It can prove to be a diagnostic challenge for physicians due to its rarity and the numerous differentials that require ruling out. Current treatment guidelines recommend the use of eculizumab to treat PNH but this treatment is quite expensive and is beyond the reach of most patients in developing countries. Moreover, the availability of treatment is also a major issue in countries like Pakistan. The term zero-sum-game is used to describe a situation where one party gains at the expense of the other [2]. Diagnosing PNH might be a triumph for physicians but simultaneously is a curse for poor patients.

We aim to present this case keeping in mind the rarity of this condition and the potentially fatal complications that can develop due to the lack of treatment.

## Case presentation

Informed consent was obtained from the patient and his family for this case report. A 28-year-old Pakistani male presented to Hayatabad Medical Complex, Peshawar in August 2017, with weakness and generalized body aches for two years, dark urine for one year, jaundice for six weeks, and intermittent low-grade fever for three weeks. The patient had been worked-up for anemia at multiple hospitals over the preceding two years but had not been diagnosed. He had no significant past medical, family or drug history and had no history of weight loss, anorexia, intravenous drug abuse, or unprotected sexual intercourse. On examination, he was vitally stable and the only clinically significant finding was pallor and jaundice. Splenomegaly could not be appreciated on examination but was visible on an abdominal ultrasound. His labs showed a hemoglobin (Hb) of 8g/dl, mean corpuscular volume (MCV) of 105fl and reticulocyte count of 4% with indirect hyperbilirubinemia (1.5 mg/dl). Peripheral smear revealed anisocytosis, hypochromic microcytosis, macrocytosis, and pencil cells. He was investigated for autoimmune and hereditary causes of anemia by direct Coombs test, red cell antibody screen, quantitative glucose-6-phosphate dehydrogenase (G6PD) levels, and osmotic fragility test, all of which came back negative. Bone marrow aspirate and trephine biopsy revealed hypercellular marrow with mainly normoblastic and a few megaloblastic changes. Qualitative assay for CD55 and CD59 was sent which came back negative, signifying the absence of these factors from the cell surface. He was subsequently labelled as a case of PNH and was prescribed eculizumab and vitamin supplements along with monthly follow-ups. He failed to follow-up and was admitted 12 months later with an ischemic stroke and aspiration pneumonia. On history taking, his family revealed non-compliance to treatment due affordability and availability issues. He died two days later due to cardiorespiratory failure.

## Discussion

The causes of anemias in our population are diverse and the workup of anemias can be very costly and cumbersome. Hemolytic anemia is a type of anemia caused by increased destruction of red blood cells (RBCs). They can be subdivided based on inheritance, structural cellular defects, point of hemolysis, and duration. Classification into extravascular and intravascular hemolytic anemias is one such subdivision based on the site of hemolysis [3]. Paroxysmal nocturnal hemoglobinuria (PNH) is a type of intravascular hemolytic anemia caused by an acquired mutation in the PIG-A (phosphatidylinositol N acetylglucosaminyltransferase subunit A) gene in hematopoietic stem cells, leading to clonal proliferation of cells deficient in a functional component of the cell membrane called glycosyl phosphatidyl inositol (GPI). GPI serves as an anchor point for proteins like CD55 (also called decay accelerating factor) and CD59 ( also called membrane inhibitor of reactive lysis) [1]. Both these proteins prevent complement-mediated RBC lysis. In the absence of anchoring points for these proteins, the RBCs are exposed to destruction by the complement system during the night when the pH of blood becomes acidic due to carbon dioxide retention. This leads to the release of hemoglobin into the blood and ultimately into the urine, causing early morning dark urine [4,5].

Complications commonly encountered in untreated PNH patients include bone marrow failure, smooth muscle dystonia, and thrombosis (as witnessed in our patient). Although venous thrombosis in PNH is well documented, there are only a few reported cases of arterial thrombosis [6]. The incidence of thrombotic events in PNH is 40% and mostly occur in large veins (cerebral, hepatic, portal, mesenteric, splenic, and renal veins). Increased incidence of arterial thrombosis has been discovered fairly recently [7]. Before the availability of eculizumab, PNH had a median survival between 10 and 22 years from the time of diagnosis, with thrombosis causing 22-67% of deaths. Twenty-nine percent to 44% of patients had at least one episode of thrombosis [8]. The mechanism of thrombosis in PNH is believed to be due to one of the following three hypotheses: platelet activation by complement which in turn causes a rise in pro-coagulant microparticles; altered plasminogen activation causing a chronic decrease in fibrinolysis; accumulation of free hemoglobin due to chronic hemolysis causing a decrease in nitric oxide production which in turn leads to endothelial dysfunction and platelet activation [9].

Current treatment guidelines recommend the use of eculizumab 600mg IV every week for four weeks, followed by 900mg IV one week later and then 900mg IV every two weeks thereafter [10,11]. Eculizumab works by decreasing hemolysis in PNH and subsequently the associated complications. It does not alter the underlying pathology of the disease and therefore must be continued for life or until spontaneous remission of the disease occurs (seen in only 12 out of 80 patients according to one study). A study in Leeds Hospital United Kingdom showed a five-year survival of 95.5% in patients receiving eculizumab treatment, compared to 66.8% in those not receiving any treatment [7]. Use of antithrombotic therapy as prophylaxis has not proven beneficial with or without the co-administration of eculizumab [9]. Eculizumab, therefore, remains the most effective therapy for this condition so far.

One of the major problems in treating PNH is the cost and availability of treatment, especially in developing countries like Pakistan. The annual household income per capita in Pakistan was 650.64 USD in 2016, whereas a single 300mg vial of eculizumab retails at around 6,819.51 USD [12,13]. This treatment is out of the reach of most of the patients in under-developed countries, therefore more effort needs to be put into developing cost-effective treatments for this condition.

## Conclusions

Paroxysmal nocturnal hemoglobinuria, although well studied, is a rarely encountered cause of hemolytic anemia and should therefore be considered when all other probable causes of hemolytic anemia have been ruled out. Price and availability of treatment is a major concern for patients as well as physicians dealing with this condition, especially in developing countries. Further research needs to be done to develop more affordable treatments for this condition.
